# Evaluation of Peripheral Zone Prostate Cancer Aggressiveness Using the Ratio of Diffusion Tensor Imaging Measures

**DOI:** 10.1155/2017/5678350

**Published:** 2017-09-26

**Authors:** Aslihan Onay, Gokhan Ertas, Metin Vural, Omer Acar, Yesim Saglican, Bilgen Coskun, Sergin Akpek

**Affiliations:** ^1^Department of Radiology, Koç University School of Medicine, Istanbul, Turkey; ^2^Department of Biomedical Engineering, Yeditepe University, Istanbul, Turkey; ^3^Department of Radiology, VKF American Hospital, Istanbul, Turkey; ^4^Department of Urology, Koç University School of Medicine, Istanbul, Turkey; ^5^Department of Pathology, Acibadem University School of Medicine, Istanbul, Turkey

## Abstract

**Purpose:**

To evaluate the aggressiveness of peripheral zone prostate cancer by correlating the Gleason score (GS) with the ratio of the diffusion tensor imaging (DTI) measures.

**Materials and Methods:**

Forty-two peripheral zone prostate tumors were imaged using DTI. Regions of interest focusing on the center of tumor foci and noncancerous tissue were used to extract statistical measures of mean diffusivity (MD) and fractional anisotroy (FA). Measure ratio was calculated by dividing tumor measure by noncancerous tissue measure.

**Results:**

Strong correlations are observable between GS and MD measures while weak correlations are present between GS and FA measures. Minimum tumor MD (MD_min_) and the ratio of minimum MD (rMD_min_) show the same highest correlation with GS (both *ρ* = −0.73). Between GS ≤ 7 (3 + 4) and GS ≥ 7 (4 + 3), differences are significant for all MD measures but for some FA measures. MD measures perform better than FA measures in discriminating GS ≥ 7 (4 + 3).

**Conclusion:**

Ratios of MD measures can be used in evaluation of peripheral zone prostate cancer aggressiveness; however tumor MD measures alone perform similarly.

## 1. Introduction

The clinical behavior of the prostate cancer is strongly correlated with Gleason score (GS) as high values of GS indicate more aggressive tumors and an increased risk of local and distant tumor spread [1]. The pretherapeutic assessment of prostate tumor aggressiveness determined by GS is crucial in personalized treatment planning. Therefore, a noninvasive evaluation tool is demanded for accurate classification of tumor aggressiveness among the GS risk groups. In detection and staging of the prostate cancer, multiparametric magnetic resonance imaging (mp-MRI) is currently the most preferable technique. An important component of the prostate mp-MRI is the diffusion weighted imaging (DWI) and the apparent diffusion coefficient (ADC) obtained from the diffusion signal data captured during imaging.

DWI employs three orthogonal gradient directions to capture diffusion signal quantified by an apparent diffusion coefficient (ADC) in the detection of cancerous prostate tumor foci and in the assessment of tumor aggressiveness. Recent studies report that the value of ADC is lower in the prostate tumor foci than the healthy prostate tissue and the tumor ADC is correlated with the tumor GS [2, 3]. Benefiting from a higher number of orthogonal gradient directions, diffusion tensor imaging (DTI) provides mean diffusivity (MD) from orientationally averaged apparent diffusivity removing the orientational dependence of the ADC in DWI [4]. In addition to this, DTI makes it possible to obtain several diffusional anisotropy measures from tissue, namely, axial, radial, and fractional anisotropy (FA). These measures may potentially provide valuable information since recent studies show that changes in the percentage volume of the diffusional anisotropic stromal tissue and of the isotropic acinal lumen space and the epithelial cells of the prostate may lead to an increased Gleason score illustrating the increased aggressiveness of the tumor [5, 6].

Current clinical applications of DTI for prostate cancer are focused predominantly on differentiation of tumor from normal prostate gland [7–13] and slightly on the assessment of tumor aggressiveness with respect to GS [14–16]. A wide range of MD and FA values from prostate tumor foci has been reported. Most of the studies show that FA of prostate tumor foci is higher than the one of noncancerous prostate tissue [7–10] while lower or equal FA values have been also reported in some studies [10–13]. The variability in DTI measures can be mainly due to the uncertainties during imaging. The use of “ratio” has been reported to minimize such uncertainties in DWI improving the performance of ADC in staging prostate cancer aggressiveness [17–21]. To the best of our knowledge, for such purpose, the MD ratio or the FA ratio from DTI has not been tested yet. In this study, we investigate the utility of MD and FA based statistical measures and the ratios of these measures to exhibit the full potential of DTI in evaluating the aggressiveness of peripheral zone prostate cancer.

## 2. Material and Methods

### 2.1. Patient Population

Consecutive patients with biopsy-proven prostate cancer who underwent MR imaging prior to the diagnosis of prostate cancer between December 2012 and December 2015 at our institute were considered for this retrospective study. Patients with benign findings or with transition zone prostate cancer or with peripheral zone prostate cancer but only having TRUS guided biopsy without complementary radical prostatectomy surgery were excluded. Patients who had peripheral zone prostate cancer proved with histopathology (using in-bore prostate biopsy or TRUS guided prostate biopsy followed by radical prostatectomy) and no contraindications to MRI were included. The institutional and research committee waived informed consent and approved this retrospective study.

A total of thirty-eight patients aged 40–77 years (mean, 64.7 years) with forty-two identified prostate cancer tumors were taken into analysis, retrospectively (in the case of multiple tumor foci, each focus of was considered separated when it was disjointed by noncancerous tissue). The tumor diameter was ranging from 5 to 55 mm (mean, 12.7 mm). Twenty-four of the tumors had radical prostatectomy while the resting tumors had in-bore prostate biopsy. A detailed demographic data is presented in Table 1.

### 2.2. MR Imaging of the Prostate and Histopathology

At our institute, prostate MR imaging has been conducted by a 3 T MR scanner (Magnetom Skyra, Siemens Medical Solutions, Erlangen, Germany) using a sixteen-channel phased array surface coil while patients were positioned still in a supine position. During imaging, to reduce motion artifacts due to bowel peristalsis, 20 mg of butylscopolamine (Buscopan; Boehringer, Germany) is administrated to the patient if tolerated. The imaging protocol includes T2-weighted imaging, dynamic contrast-enhanced imaging (DCE-MRI), and diffusion weighted imaging to validate mp-MRI. However, for the current study, the protocol is extended to incorporate diffusion tensor imaging (DTI). Average acquisition time for mp-MRI is 30 minutes and additional time for DTI is around 3 minutes.

Triplanar T2-weighted images are acquired using a turbo spin-echo imaging sequence with 100 ms echo time (TE), 3566–3631 ms repetition time (TR), 512 × 352 matrix size, 200 mm field of view (FOV), and 3 mm slice thickness. DTI is performed in the axial plane using a 2D single shot echo-planar imaging sequence (EPI) in 12 gradient directions at two different *b*-values (*b* = 0 and 800 s/mm^2^) with the following parameters: 77–81.3 ms TE, 3200 ms TR, 128 × 106 matrix size, 260 × 215 mm^2^ FOV, 3.6 mm slice thickness, and 1 × 1 × 3.6 mm^3^ interpolated voxel size.

Acquired images of mp-MRI are evaluated and each identified lesion is scored according to the PIRADS v2 classification system from ESUR. For a lesion having a PIRADS score ≥ 4, either in-bore prostate biopsy or TRUS guided prostate biopsy followed by radical prostatectomy is scheduled. The mean time interval between imaging and each procedure is 73 days and 34 days, respectively. In the case of radical prostatectomy, the specimens are fixed in 10% buffered neutral formalin and the surgical margins are painted with ink. Prostate is serially cut into 3-4 mm sections in a plane perpendicular to the long axis of the prostate (from apex to base). Each slice is sequentially submitted in total for routine tissue processing and as whole mount sectioning. Routine sections are stained with hematoxylin and eosin.

All tumor foci are marked on 16-sector divided standardized prostate diagram by a pathologist. The pathologist is blind to any MRI interpretations and evaluates all tumor foci according to its Gleason score. In the case of multiple tumor foci separated by noncancerous tissue, each focus is considered separately. The GS of every foci is categorized into four risk groups: low (GS = 6), intermediate-low (GS = 7 (3 + 4)), intermediate-high (GS = 7 (4 + 3)), and high (GS = 8–10) risk cancers. Tumor sizes, PSA levels, and final tumor stages for those GS based groups are as reported in Table 2.

### 2.3. Image Evaluation and ROI Placement

**Figure 1:**
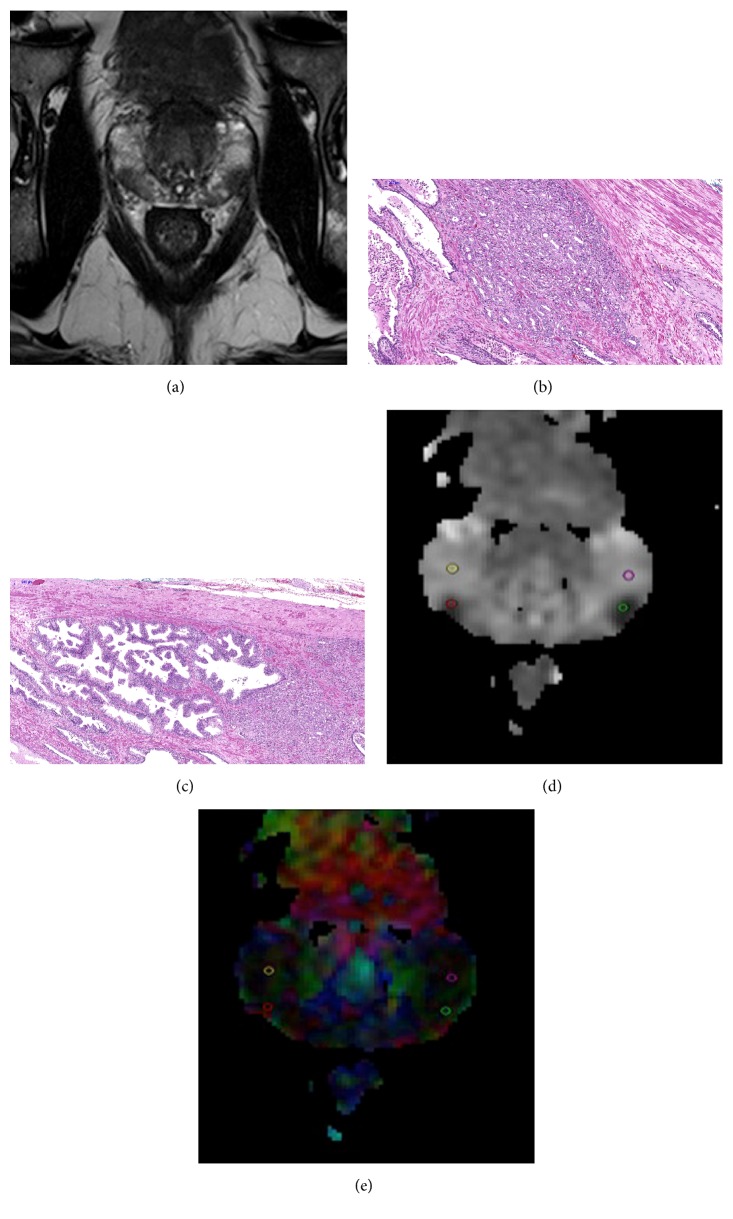
A 62-year-old patient with two prostate cancer tumor foci in the right and the left peripheral zones both with GS7 (4 + 3). (a) Representative T2-weighted image slice, (b, c) histopathology slices confirming prostate cancer, and (d) MD and (e) FA maps with the ROIs placed for the two tumor foci (solid red line and green line contours) and for the noncancerous tissue, respectively (solid yellow line and purple line contours).

T2-weighted images and diffusion tensor images were transferred to a workstation for subsequent analysis. The images were evaluated by two radiologists (MV and AO with 7 and 2 years of experience in prostate mp-MRI, resp.) and a pathologist (YS with 10 years of experience in urological pathology) using Syngo MR D13C software (Siemens Healthcare) installed on the workstation. T2-weighted images providing better anatomical orientation were used to guide the radiologists while localizing the tumors. When localized, a tumor was evaluated on the relevant MD map of DTI further to match the tumor focus considering the standardized histopathologic diagrams as reference while taking alterations in the prostate shape and size caused by preservation of the specimen into account. A tumor focus was assumed to be matched when it was in the same region on both histopathology and MRI. After matching, a region of interest (ROI) was manually placed on the relevant MD map with care to include only the center of tumor foci. An additional “reference” ROI with similar size as the tumor ROI was placed for the noncancerous tissue in the same prostatic region. All ROIs were circular with 3 mm diameter and placed by the two radiologists by mutual agreement. Both radiologists and the pathologist were blinded to Gleason score during image evaluation and ROI placement. Using the same software, the ROI pairs placed on the MD maps were automatically reproduced on the FA maps of DTI (see Figure 1).

DTI measures determined by the software for each ROI were recorded. These measures included the minimum, the maximum, the mean, and the standard deviation for MD, as well as for FA. The mean (*μ*) and the standard deviation (*σ*) measures were used to assess contrast-to-noise ratio (CNR) for both MD and FA maps. The CNR was calculated by

(1) $$\begin{array}{c} CNR=\frac{\mu_{tumor}-\mu_{tissue}}{\sqrt{\sigma_{tumor}^{2}+\sigma_{tissue}^{2}}}. \end{array}$$

The minimum, the maximum, and the mean measures from the tumor and from the noncancerous tissue were used to calculate the ratio of measures.

## 3. Statistical Analysis

Systematic differences in the DTI measures between the tumor and the noncancerous prostate tissue were tested using Wilcoxon signed-rank test. Spearman correlation coefficient (*ρ*) was used to assess any correlation between DTI measures including the measure ratios and GS. Systematic differences in DTI measures (including the ratios of the measures) between GS ≤ 7 (3 + 4) and GS ≥ 7 (4 + 3) risk group tumors were tested using a Mann–Whitney *U*-test. Receiver operating characteristic (ROC) curves were plotted and the areas under the ROC curves (AUC) were used as indices of performance for each measure in discriminating GS ≥ 7 (4 + 3) from GS ≤ 7 (3 + 4) risk group tumors. Statistical analyses were performed using SPSS software for Windows (v23.0; Chicago, IL). *P* < 0.05 was considered statistically significant.

## 4. Results

DTI measures captured for all prostate tumor foci and for all noncancerous tissue are as seen in Table 3. MD based DTI measures from the tumors are smaller than the ones from the noncancerous prostate tissue and the differences are all significant (*P* < 0.05). On the other hand, FA based DTI measures from the tumors are higher than the ones from the noncancerous prostate tissue significantly (*P* < 0.05). These results verify the appropriate placement of the ROI for the noncancerous prostate tissue. Average contrast-to-noise ratio is calculated as 16.0 from MD maps and 3.1 from FA maps showing that MD maps provide better tumor contrast with respect to FA maps of DTI.

**Figure 2:**
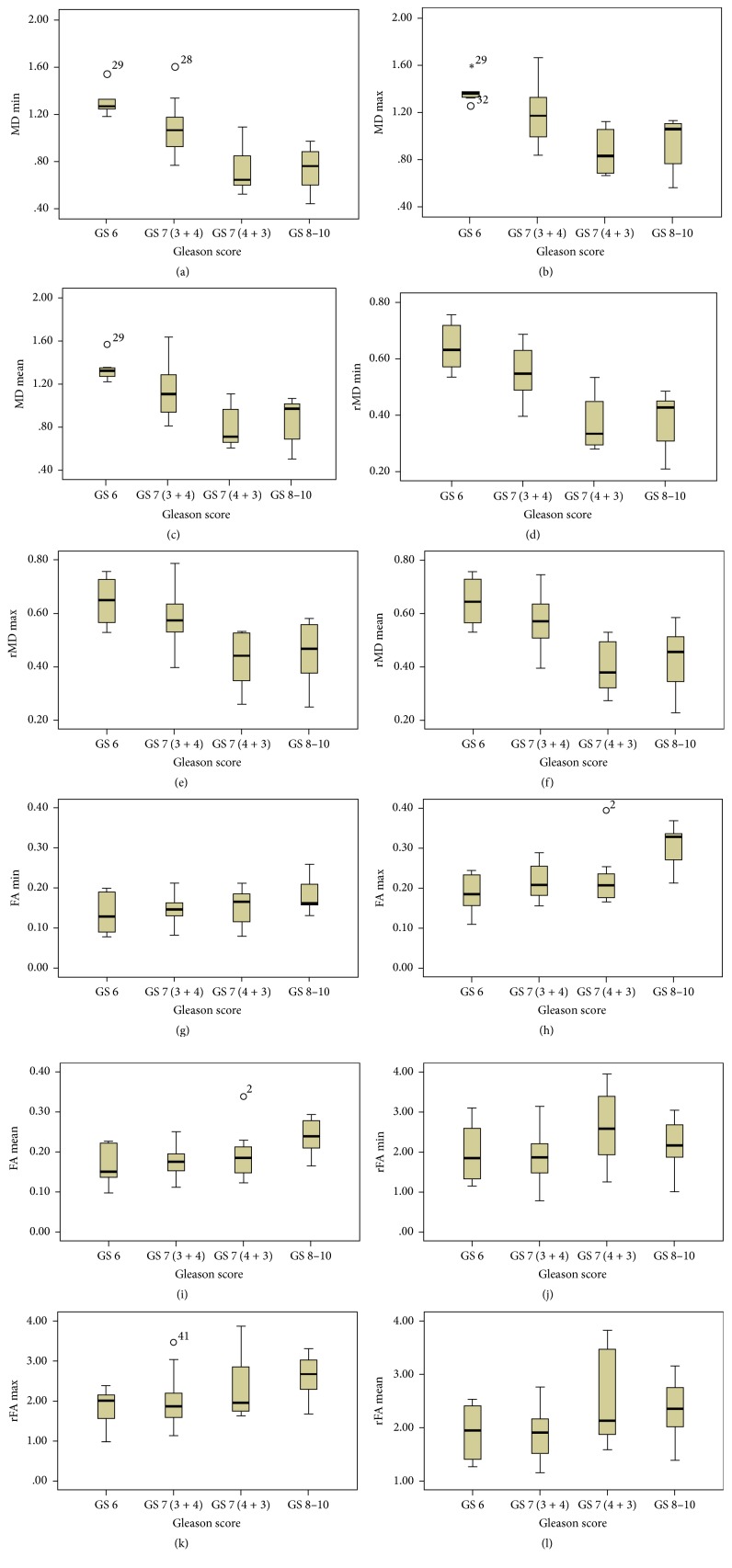
Box plots of (a–f) MD and (g–l) FA measures stratified by Gleason score groups (MD in 10^−3^ mm^2^/s).

Box plots of MD and FA based DTI measures for all tumors stratified by GS groups are shown in Figure 2. Correlations determined between the DTI measures and the Gleason score are given in Table 4. Strong negative correlations are observable between the Gleason score and all MD based tumor DTI measures including the ratios (*ρ* = −0.73 to −0.60, *P* < 0.001). On the other hand, FA based DTI measures provide weak positive correlations with Gleason score. Among all DTI measures, MD_min_ has the highest correlation with GS (*ρ* = −0.73) while rMD_min_ is correlated with GS in a similar manner (*ρ* = −0.73).

**Figure 3:**
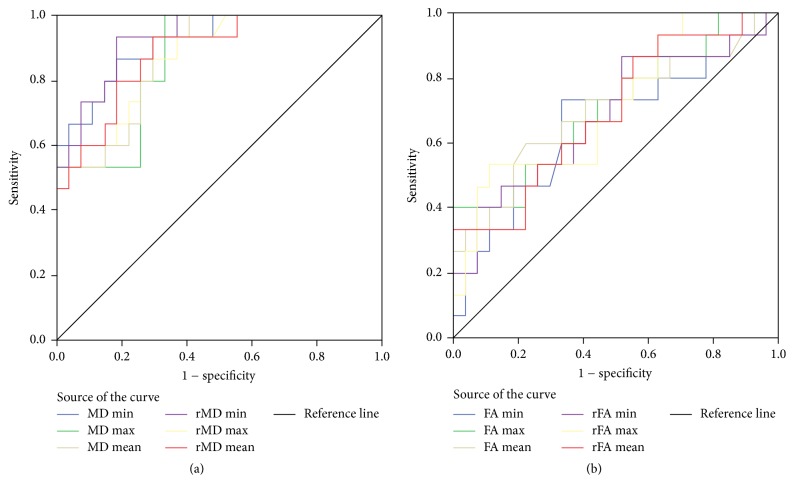
ROC curves of (a) MD and (b) FA measures in discriminating GS ≥ 7 (4 + 3) from GS ≤ 7 (3 + 4) risk group tumors.

Case summaries of the measures for GS ≤ 7 (3 + 4) and GS ≥ 7 (4 + 3) risk group tumors are as seen in Table 5. Smaller values for MD measures and larger values for FA measures are the precursors of GS ≥ 7 (4 + 3). Between GS ≤ 7 (3 + 4) and GS ≥ 7 (4 + 3), systematic differences are present for all MD based measures. However, when FA based measures are considered, the differences are significant for FA_max_, FA_mean_, rFA_max_, and rFA_mean_ only (*P* < 0.05). The results of the ROC analyses of the measures in discriminating GS ≤ 7 (3 + 4) from GS ≥ 7 (4 + 3) risk group tumors are shown in Table 6. MD based measures (AUCs = 0.93–0.86) perform quite better than the FA based measures (AUCs = 0.71–0.70) (see Figure 3). FA measures show almost the same low performance. Among all DTI measures, rMD_min_ stands out in terms of its highest performance (AUC = 0.93); however MD_min_ shows almost the same performance (AUC = 0.92).

## 5. Discussion

Diffusion tensor imaging (DTI) provides mean diffusivity (MD) and fractional anisotropy (FA) that reflect molecular diffusion rate and diffusional anisotropy characteristics as sensitive measures of altered tissue structure. In this study, we investigate the utility of statistical measures (i.e., the minimum, the mean, and the maximum) based on MD and FA and the ratios of these measures to exhibit the full potential of DTI in evaluating the aggressiveness of peripheral zone prostate cancer.

The normal supporting stroma and capsule of the prostate consist of dense fibroelastic connective tissue in which prostate glands are dispersed. At the central zone of the prostate, these glands increase greatly in size as a normal part of the aging process. Conversely, the peripheral zone is the part of the prostate that most of the cancers are located. Normal peripheral zone prostate tissue is composed of three gland component volumes including stroma, epithelium, and lumen space. In the case of prostate cancer, the percentage volume of stromal tissue decreases because of the collection of small atypical glands. As GS and tumor grade increase, tumor cells either form cribriform, fused glands, and solid sheets or infiltrate as individual cells without forming luminal spaces [5]. Although the diffusional anisotropy characteristics of peripheral prostate tissue are still under discussion, it is known that while the lumen space and the epithelium show anisotropic diffusion, the diffusion is isotropic within the stroma. The varying percentage volumes of the isotropic and the anisotropic diffusional compartments are expected to be associated with the measurable differences on FA value corresponding to tumor aggressiveness [6].

Our results from 38 patients with 42 identified prostate cancer tumors demonstrate that strong negative correlations are observable between GS and MD based tumor DTI measures in agreement with [14–16] and the ratios of these measures. On the other hand, our results show weak positive correlations or no significant correlations between GS and FA based DTI measures including the ratios. Beside, contradicting correlations between GS and FA have been reported in the literature: strong positive correlation [14], weak correlation with a very low value of the correlation coefficient [15], or no significant correlation [16]. Among all measures studied, the ratio of minimum MD from prostate tumor foci and noncancerous PZ prostate tissue shows the highest correlation with GS. Though, the minimum MD from prostate tumor foci alone shows almost the same correlation.

In discriminating GS ≤ 7 (3 + 4) from GS ≥ 7 (4 + 3) risk group tumors, our results show that systematic differences are present for all MD based measures as expected considering the strong negative correlations with GS. Among the FA based measures, the differences are present only for maximum tumor FA, mean tumor FA, ratio of maximum FA, and ratio of mean FA although these FA measures are correlated weakly with GS. MD based measures perform quite better than the FA based measures. The ratio of minimum MD from prostate tumor foci and noncancerous PZ prostate tissue stands out in terms of its highest discrimination performance. However, the minimum MD from prostate tumor foci alone provides almost the same remarkably high performance.

Ratios of DTI measures tested in this study show similar performances of the measures alone in correlations with GS and in discriminating GS groups. While calculating the ratios, noncancerous peripheral zone prostate tissue is considered as the reference tissue. Distinguishable performance differences can be obtained when noncancerous transition zone prostate tissue, urinary bladder tissue, or urine is used as reference tissue. These tissues have been tested in DWI to improve the performance of ADC [17–21]). On the other hand, similarities between the performances of the measures and the ratios of the measures may be due to uncertainties in human physiology or limitations of the MR imaging technique. FA and MD of the noncancerous PZ tissue may demonstrate negative and positive correlations with age, respectively [22]. The noncancerous PZ tissue may be hindered by some small tumor foci of a multicentric tumor left undetected by MRI due to the limited spatial resolution of the imaging protocol. There may also be chronic inflammation or fibrosis left undetected by MRI but present within the noncancerous PZ tissue [18].

There are some limitations of the study. Histopathologic specimens of the tumors analyzed in this study were obtained from either prostatectomy or in-bore biopsy. The whole mount histologic specimens from prostatectomy reflect the tumor with highest accuracy. However, type of utilization may cause selection bias since radical prostatectomy material cannot be utilized from the patients with low-risk tumor on TRUS-bx included in active-surveillance regimens or the patients with high-risk disease and being candidates for hormone or radiation therapy. In-bore biopsy cases of this study prevent the possible selection bias. In in-bore biopsy, a direct match between the identified tumor and the corresponding histopathologic specimen can be obtained with good accuracy since the biopsy needle is targeted to the tumor under the guidance of MR device.

The diffusion tensor imaging protocol of this study employs 12 diffusion encoding directions, two different* b*-values (i.e., *b* = 0 and 800 s/mm^2^), and a voxel size of 3.6 mm^3^. More robust estimations for DTI measures that may lead to stronger correlations with GS especially for FA based measures can be obtained by using a larger number of diffusion encoding directions and *b*-values [14]. However, use of a larger number of diffusion encoding directions or *b*-values would result in an increase in the scanning time that may lead to severe motion artifact while making the imaging unfeasible for clinical practice. On the other hand, larger number of diffusion encoding directions may lead to lower values for FA measures [23]. The voxel size is small enough to avoid any possible bias on DTI measures due to partial volume averaging. The use of a larger voxel size may lead to decreased FA measures due to partial volume averaging especially in the presence of heterogeneously oriented prostate stromal smooth muscle [24].

In the current study, small ROIs have been used and T2-weighted images have been evaluated to identify the fibrous prostate tissue during the placement of the ROIs. Selection of the ROI size and the placement of the ROI require utmost attention for good repeatability and reliability for the DTI measures. Large differences in the measures can be experienced when the ROI size is larger or when the ROI is placed on fibrous tissue of the prostate gland.

The signal-to-noise ratio measured from the DTI images acquired with *b* = 0 s/mm^2^ during this study is equal to 58 on average. This value is far beyond the minimum SNR recommended (i.e., 20) to obtain unbiased DTI measures [25]. In addition to this, it has been reported that a higher SNR level may lead to more accurate diffusion and also anisotropy estimates especially for cancerous prostate tissue while it is less effective for noncancerous prostate tissue [12, 15].

This work is focused on the clinical benefits of the DTI measures and of the ratio of these measures in assessment of peripheral zone prostate cancer aggressiveness. In the near future, we plan to assess the utility of noncancerous transition zone prostate tissue, urinary bladder tissue, and urine as reference tissue in ratio calculations and we plan to develop an automated method to obtain ROIs for the tumor foci and for the reference tissue. We also plan to extend our study to include the translational zone prostate cancer.

In conclusion, the ratio of MD based diffusion tensor imaging measures can be used to determine the aggressiveness of peripheral zone prostate cancer; however, a similar diagnostic performance may be obtained by using the tumor MD measure alone.

## Figures and Tables

**Table 1:** Detailed demographic data.

Total number of patients	38
Age (years), mean (range)	64.7 (40–77)
PSA (ng/mL), mean (range)	9.0 (4–72)
Tumor diameter (mm), mean (range)	12.7 (5–55)
Total number of tumors	42
Number of tumors with in-bore biopsy	18
Number of tumors with	
cT2a	5
cT2b	5
cT2c	3
cT3a	3
cT3b	2
Number of tumors with radical prostatectomy	24
Number of tumors with	
pT2a	4
pT2b	0
pT2c	14
pT3a	4
pT3b	2
Number of tumors with	
GS6	6 (14%)
GS7 (3 + 4)	21 (50%)
GS7 (4 + 3)	8 (19%)
GS8–10	7 (17%)

**Table 2:** Tumor sizes, PSA levels, and final tumor stages stratified by Gleason score groups.

	*n*	Tumor size (mm)	PSA level (ng/mL)	Final tumor stage
GS6	6	8.0 ± 2.4^a^	5.9 ± 1.2	cT2a (2) ^b^
				pT2c (4)
GS7 (3 + 4)	21	10.6 ± 4.2	6.5 ± 2.0	cT2a (1), cT2b (3), cT2c (1)
				pT2a (3), pT2c (9), pT3a (3), pT3b (1)
GS7 (4 + 3)	8	12.6 ± 5.0	9.2 ± 6.0	cT2a (2), cT2b (1), cT2c (2), cT3a (1)
				pT2a (1), pT2c (1)
GS8–10	7	23.0 ± 17.6	19.0 ± 23.8	cT2b (1), cT3a (2), cT3b (2)
				pT3a (1), pT3b (1)

^a^Mean ± SD. ^b^Number of tumors.

**Table 3:** DTI measures from the tumor foci and from the noncancerous prostate tissue.

	Tumor foci	Noncancerous tissue
MD_min_	0.98 ± 0.28^a^	1.97 ± 0.25
	(0.44–1.60)^b^	(1.36–2.47)
MD_max_	1.11 ± 0.27	2.08 ± 0.26
	(0.50–1.53)	(1.54–2.55)
MD_mean_	1.05 ± 0.27	2.02 ± 0.25
	(0.50–1.63)	(1.51–2.50)
FA_min_	0.15 ± 0.04	0.08 ± 0.03
	(0.08–0.26)	(0.03–0.16)
FA_max_	0.23 ± 0.06	0.11 ± 0.04
	(0.11–0.40)	(0.06–0.25)
FA_mean_	0.19 ± 0.05	0.09 ± 0.03
	(0.10–0.34)	(0.04–0.22)

^a^Mean ± SD. ^b^Minimum–maximum and MDin 10^−3^ mm^2^/s. All statistical values are significant (*P* < 0.05).

**Table 4:** Correlations between DTI measures and Gleason score (*ρ*_GS_).

	*ρ*_GS_
MD_min_	−0.73
rMD_min_	−0.73
MD_mean_	−0.67
rMD_mean_	−0.64
MD_max_	−0.63
rMD_max_	−0.60
FA_max_	0.44
FA_mean_	0.41
rFA_max_	0.35
FA_min_	0.31
rFA_mean_	0.29
rFA_min_	0.23

Statistical values are significant for all MD measures and for FA_max_, FA_mean_, and rFA_max_  (*P* < 0.05).

**Table 5:** Tumor DTI measures from GS ≤ 7 (3 + 4) and GS ≥ 7 (4 + 3) risk group tumors.

	GS ≤ 7 (3 + 4) *n* = 27	GS ≥ 7 (4 + 3) *n* = 15
MD_min_	1.12 ± 0.21^a^	0.73 ± 0.19
	(0.77–1.60)^b^	(0.44–1.09)
MD_max_	1.23 ± 0.23	0.90 ± 0.20
	(0.84–1.66)	(0.57–1.13)
MD_mean_	1.18 ± 0.21	0.82 ± 0.20
	(0.81–1.63)	(0.50–1.11)
FA_min_	0.14 ± 0.03	0.17 ± 0.05
	(0.08–0.21)	(0.08–0.26)
FA_max_	0.21 ± 0.05	0.26 ± 0.08
	(0.11–0.29)	(0.17–0.40)
FA_mean_	0.17 ± 0.04	0.21 ± 0.06
	(0.10–0.25)	(0.12–0.34)
rMD_min_	0.57 ± 0.09	0.37 ± 0.10
	(0.39–0.76)	(0.21–0.53)
rMD_max_	0.59 ± 0.09	0.44 ± 0.11
	(0.40–0.79)	(0.25–0.58)
rMD_mean_	0.58 ± 0.09	0.41 ± 0.11
	(0.39–0.75)	(0.23–0.58)
rFA_min_	1.90 ± 0.60	2.79 ± 1.60
	(0.78–3.15)	(1.01–6.84)
rFA_max_	1.93 ± 0.56	2.57 ± 0.94
	(0.97–3.47)	(1.64–4.91)
rFA_mean_	1.92 ± 0.49	2.58 ± 1.02
	(1.15–2.75)	(1.38–5.09)

^a^Mean ± SD. ^b^Minimum–maximum and MDin 10^−3^ mm^2^/s. All statistical values are significant (*P* < 0.05) except for FA_min_ and rFA_min_.

**Table 6:** Diagnostic performance of DTI measures in discriminating GS ≤ 7 (3 + 4) from GS ≥ 7 (4 + 3).

	AUC	Std. error	95% confidence interval of AUC	
			Lower bound	Upper bound
rMD_min_	0.93	0.05	0.85	1.00
MD_min_	0.92	0.04	0.83	1.00
rMD_mean_	0.88	0.05	0.78	0.99
MD_mean_	0.88	0.05	0.77	0.98
rMD_max_	0.87	0.06	0.76	0.98
MD_max_	0.86	0.06	0.75	0.97
rFA_max_	0.71	0.08	0.55	0.88
FA_max_	0.71	0.09	0.54	0.88
FA_mean_	0.70	0.09	0.53	0.88
rFA_mean_	0.70	0.09	0.53	0.86
